# Jelleine-I Membrane Interaction-related Biological Properties and Antimicrobial Activity against MDR, XDR, and PDR-*Acinetobacter baumannii* Clinical Isolates

**DOI:** 10.1021/acsomega.4c09073

**Published:** 2025-03-11

**Authors:** Adrielle Pieve de Castro, Julio Cesar Moreira Brito, Wanderson Aparecido Brandão Candido, Amanda Souza Félix, Rodrigo Moreira Verly, Jarbas Magalhães Resende, Letícia Lopes-de-Souza, Carlos Chávez-Olórtegui, Simone Odília Antunes Fernandes, Valbert Nascimento Cardoso

**Affiliations:** 1 Laboratório de Radioisótopos, Departamento de Análises Clínicas e Toxicológicas, Faculdade de Farmácia, Universidade Federal de Minas Gerais, Campus Pampulha, Belo Horizonte, Minas Gerais 31270-901, Brazil; 2 Fundação Ezequiel Dias; Diretoria de Pesquisa e Desenvolvimento, Belo Horizonte, Minas Gerais 30510-010, Brazil; 3 Departamento de Química, Faculdade de Ciências Exatas, Universidade Federal dos Vales do Jequitinhonha e Mucuri, Diamantina, Minas Gerais 39100-000, Brazil; 4 Departamento de Química, Instituto de Ciências Exatas, Universidade Federal de Minas Gerais, Belo Horizonte, Minas Gerais 31270-901, Brazil; 5 Departamento de Bioquímica e Imunologia, Instituto de Ciências Biológicas, Universidade Federal de Minas Gerais, Belo Horizonte, Minas Gerais 31270-901, Brazil

## Abstract

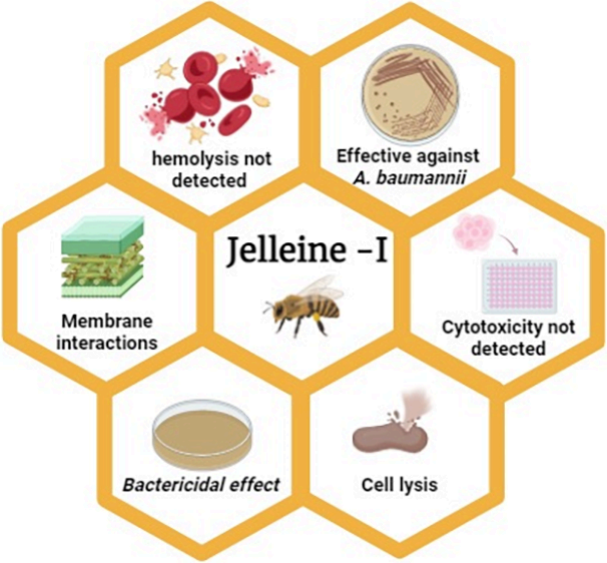

Emerging bacterial infections pose a serious threat to human health. *Acinetobacter baumannii* is a particular concern due to its antimicrobial resistance phenotypes, especially to carbapenems. In this context, antimicrobial peptides appear as a promising class. Jelleine-I is a peptide identified from the royal jelly from *Apis mellifera* bee, which has demonstrated significant antibacterial effects against various microorganisms. This study aimed to characterize the activity of jelleine-I against clinical isolates of *A. baumannii* resistant to carbapenems (CRAB) and with different resistance phenotypes, in addition to investigating the peptide–membrane interaction in biomimetic media. Microbiological assays with jelleine-I performed against *A. baumannii* with MIC values of 8–16 μM were observed. Biophysical studies on the bacterial mimetic membrane show a possible disruption of the organization of the phospholipid bilayer. The significant affinity promoted by entropic and enthalpic contributions suggests that the main antimicrobial action occurs on the bacterial membrane. In addition, the negligible hemolytic activity and toxicity against VERO and HaCaT cells reveal jelleine-I as a potential novel antimicrobial agent, especially against microorganisms that exhibit high and diverse antimicrobial resistance, such as *A. baumannii*.

## Introduction

In recent decades, multidrug-resistant (MDR; resistance to at least three classes of antimicrobials) pathogens have become an urgent problem in clinical anti-infective treatment and pose a major threat to the health and lives of people worldwide. According to the World Health Organization (WHO), 700,000 deaths worldwide are attributed to antimicrobial resistance (AMR) each year, with an estimated 10 million deaths annually caused by resistant bacterial infections by 2050 if the current trend of AMR development is not suppressed.^1,2^

In this scenario, infections caused by Gram-negative bacteria have become increasingly difficult to treat or even untreatable, particularly due to the presence of an outer membrane and its composition of phospholipids/lipopolysaccharides. These properties play an important role in favoring resistance phenotypes and consequently protection against most of the available conventional antimicrobials.^3,4^ Among Gram-negative bacteria, MDR-*Acinetobacter baumannii* is responsible for a significant proportion of healthcare-associated complications worldwide, mainly affecting immunocompromised individuals, especially frequently in the intensive care unit.^5^

Carbapenem antibiotics such as imipenem, meropenem, doripenem, but not ertapenem, are considered valuable treatment options for MDR-*A. baumannii* infections. However, a steady increase in the number of carbapenem-resistant strains of *Acinetobacter baumannii* (CRAB) has been recorded. In addition, the phenotypes of extensive resistance (XDR; MDR plus resistance to carbapenems) and pan-resistance (PDR; XDR plus resistance to polymyxins) stand out among *A. baumannii* isolates.^6−8^ This critical panorama has led the WHO to classify CRAB as a critical pathogen causing life-threatening infections with the highest priority for research and development of new antibiotics, with the aim of controlling the rapid and alarming advance of this bacterium in hospital environments.^9^

In this context, antimicrobial peptides (AMPs) are considered as alternatives to the conventional antimicrobial agents, mainly due to their promising mechanism of non- specific physical targeting to the cell membrane and disrupting it to sterilize microbes,^10^ thus hampering the development of bacterial resistance.^11,12^

Jelleine-I is a peptide that was originally isolated from the royal jelly of bees (*Apis mellifera*). It consists of a short sequence of eight amino acid residues with a C-terminal carboxyamide (PFKISIHL-NH2). This peptide exhibits excellent antimicrobial activity against Gram-positive, and Gram-negative bacteria as well as yeast.^13^ In this study, we synthesized jelleine-I and investigated its membrane interaction, toxicity and efficacy as an antibacterial agent against clinical isolates of CRAB, MDR-*Acinetobacter baumannii*, XDR-*Acinetobacter baumannii* and PDR-*Acinetobacter baumannii*.

## Results and Discussion

### Synthesis and Purification of Jelleine-I

Jelleine-I was synthesized in the Peptide Synthesis and Structure Laboratory (LASEP/UFMG) and presented gross yield (before purification) of approximately 50% (150 mg). After purification, a purity of 97.48% was obtained. The peptide was collected and evaluated in MALDI-TOF (Figure S1). After purification by RP-HPLC, the product was lyophilized, yielding a final recovery of approximately 81.8%.

### Spectrometric Characterization

The primary structure of jelleine-I was characterized by mass spectrometry and NMR spectroscopic. The peak of protonated jelleine-I is observed in the MALDI-ToF-MS spectrum of the peptide, which sequence was confirmed using MS/MS analysis (Figure S2). The spin systems of all residues were recognized in the NMR spectra of jelleine-I in DMSO-*d*6. All ^1^H and ^15^N resonances of the amide groups are observed in the ^1^H–^15^N Heteronuclear Single Quantum Coherence (HSQC) spectrum (Figure S3). The Hα-Cα correlations of all residues are observed in the respective ^1^H–^13^C HSQC spectrum (Figure S4). The characteristic spin systems of the aliphatic side chains were detected from the amide ^1^H chemical shift of each amino acid residue in the respective Total Correlation Spectroscopy (TOCSY) spectrum (Figure S5). Nuclear Overhauser Spectroscopy (NOESY) allowed the unequivocal assignment of Ile-4 and Ile-6 through sequential inter-residue correlations (Figure S5).

### Isothermal Titration Calorimetry (ITC)

**Figure 1:**
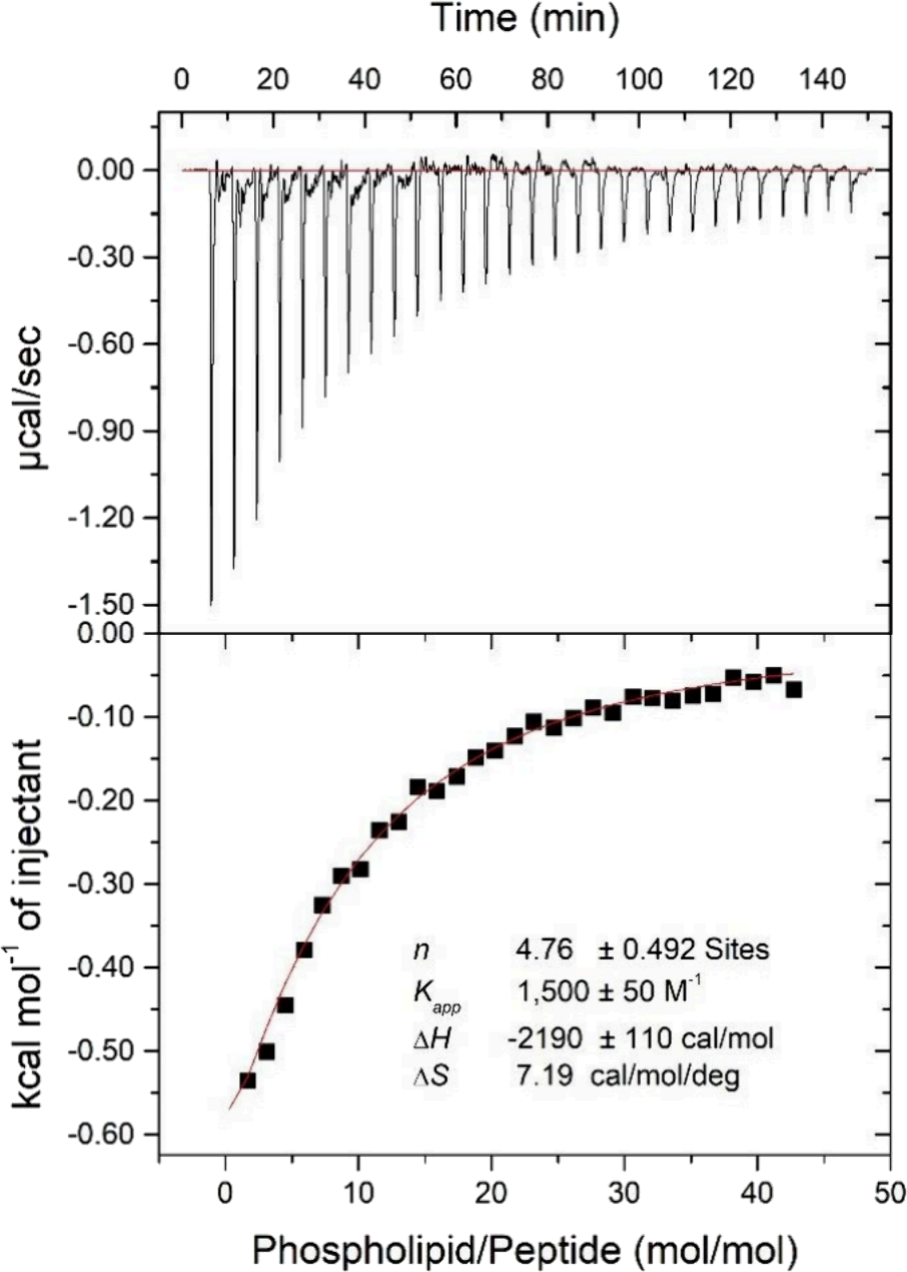
Heat flow as a function of time and respective curve with isotherm adjustment for the titration of 25 μM jelleine-I with 20 mM POPC:POPG (3:1).

ITC assays were performed by titrating 20 mM POPC (2-oleoil-1-pamlitoil- sn - glicerol-3-fosfocolina) and POPG (2-oleoil-1-pamlitoil- sn -glicerol-3-glicerol) - POPC:POPG (3:1) LUVs in 25 μM jelleine-I solution. LUVs solutions were titrated into the peptide solution, to avoid heat of peptide aggregation process during the experiment.^14,15^Figure 1 (upper panel) presents the heat flow for investigate the membrane interaction of jelleine-I in an anionic vesicular medium, where each peak reflects the heat flow produced by the injection of 5 μL of vesicles into the peptide solution.

The thermodynamic parameters of the peptide-membrane interaction were determined from the isotherm fitting (Figure 1, lower panel). The apparent constant (*Kapp*) in the order of 1.50 × 10^3^ L.mol^–1^, indicates a medium-to-strong peptide-membrane interaction. The entropic factor (-*T*Δ*S* = 2.14 × 10^–3^ cal.mol^–1^) is equivalent to the enthalpy (Δ*H* = −2.19 × 10^–3^ cal.mol^–1^), revealing a balance between entropic and enthalpic contributions. Whereas the negative enthalpy indicates a predominance of exothermic interactions as a result of electrostatic interactions between the cationic peptide and anionic vesicles,^15^ the peptide-membrane process leads to the desolvation of both the external surface of the LUVs and the hydrophilic region of the peptide chain. Therefore, a greater number of free water molecules in the system increases the entropic components associated with translation and rotation of water molecules.^15,16^

### Surface Plasmon Resonance (SPR)

**Figure 2:**
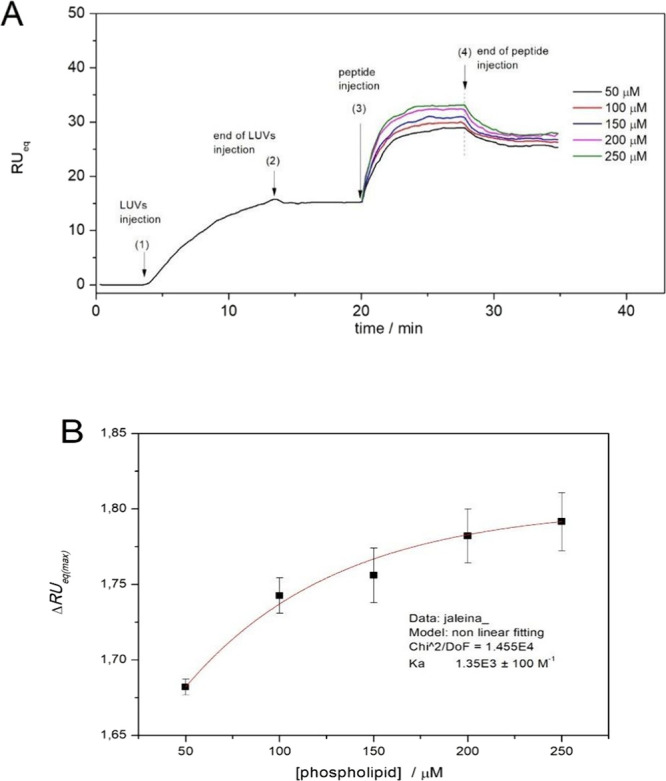
SPR Sensograms for the (A) interaction of 10 μM jelleine-I and POPC:POPG LUVs (50–250 μM) immobilized on the surface of the DTT-gold sensor chip. The numbers correspond to (1) start of injection of LUV, (2) end of LUV injection, (3) start of peptide injection, and (4) end of peptide injection. (B) Dependence of RUeq(max) intensities after 12 min of jelleine-I injection on lipid concentration.

To investigate the affinity of jelleine-I in the presence of anionic membranes, SPR experiments were carried out by injecting peptide onto POPC:POPG (3:1) LUVs immobilized on a SiO2 sensor chip in the presence of Tris-HCl running buffer. The sensograms profiles of the peptide-membrane interaction show significant increases in the RU signal with addition of peptide (Figure 2). The peptide-membrane interaction resulted in an increase in RU signal intensity proportional to the phospholipid concentration.^16^ Similar results were achieved in another cationic peptide, LyeTxI-b, which also showed activity against isolates of *A. baumannii* resistant to carbapenems with low minimal inhibitory concentration (MIC) values (1 μM).^17^ The surface partition coefficients (*K*) for the interactions between the peptide and the anionic membrane were determined through simulations based on eq 1, where the RU signal intensity was analyzed as a function of phospholipid concentration (Figure 2B), resulting in *K* of 1.35 × 10^3^ ± 100 M^–1^.

### Hydrodynamic Diameter (Dh) and Zeta Potential (ζ- Potential)

**Figure 3:**
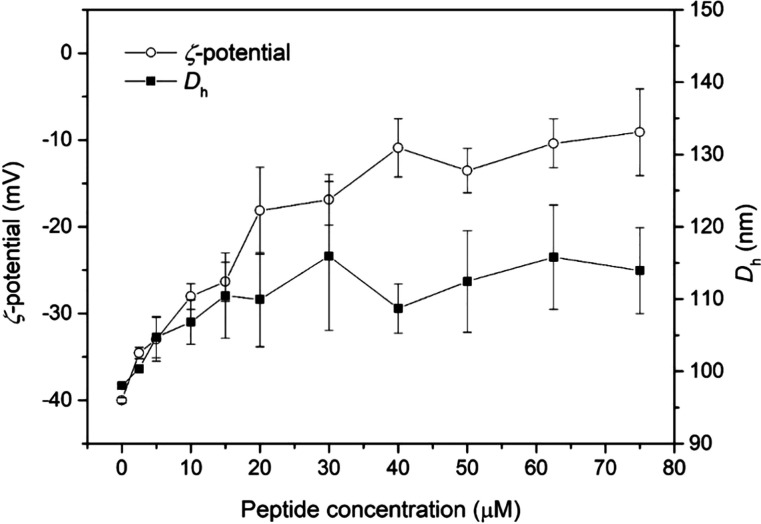
Hydrodynamic diameter (*D*h) and normalized zeta potential (ζ-potential) of 500 μM POPC:POPG LUVs with addition of jelleine-I.

The peptide-membrane interaction can disturb the organization of phospholipid bilayer and, consequently, it alters the volume and surface charges of the phospholipid vesicles.^18−20^ Therefore, measurements of the hydrodynamic diameter (*D*h) and zeta potential (ζ*-*potential) of POPC:POPG (3:1) LUVs (∼98 nm) were performed before and after the addition of jelleine-I (Figure 3). The interaction of jelleine-I with anionic LUVs leads to an increase in both *D*h and ζ*-*potential values of phospholipid vesicles until a plateau is reached. The maximum *D*h acquired by the LUVs in the presence of the jelleine- I is obtained at 30 μM of peptide concentration. Interestingly, at peptide concentration ≥40 μM, the polydispersity index (PDI) overcame 0.3, indicating a heterogeneous size distribution of the vesicles. This suggests stronger membrane-disruptive properties for the peptide, in accordance with affinity constant determined in the SPR experiments. For the electrophoretic mobility, the initial values of ζ-potential are near to −40 mV and the maximum of ζ potential takes place at a peptide concentration near 40 μM, as a consequence of the electrostatic interaction between jelleine-I and negative lipid bilayers.^20,21^ These results are in line with the negative enthalpic contribution of peptide- membrane interaction, determined from ITC experiments.

### Jelleine-I *In Vitro* Antibacterial Activity against *A. baumannii*

#### Minimal Inhibitory Concentration (MIC) and Minimal Bactericidal Concentration (MBC) Evaluation

The antibacterial activity of jelleine-I and the control against clinically relevant drug-resistant *A. baumannii* strains was assessed by determining the MICs and MBCs. Testing new antimicrobial candidates against clinical strains of *A. baumannii* is essential due to the considerable interstrain variability and the impact of growth conditions on antimicrobial susceptibility, which are key factors in addressing multidrug-resistant (MDR) infections.^22^*A. baumannii* is a highly adaptable pathogen known for its ability to acquire resistance to multiple antibiotics, making it a critical target for new drug development and interstrain variability can significantly impact the evaluation of antimicrobial efficacy.^22^ Standardized strains used in preclinical testing may not represent the genetic and phenotypic diversity found in clinical isolates, potentially leading to an overestimation or underestimation of a drug’s effectiveness. Incorporating clinical strains in preclinical testing can lead to the development of more robust and effective antimicrobial therapies.^23^ This approach not only enhances the predictive value of preclinical studies but also aids in the identification of potential resistance mechanisms and the optimization of treatment regimens for resistant infections.^22,23^ As shown in Table 1, jelleine-I exhibited activity against all tested *A. baumannii* isolates, with MICs ranging from 8 to 32 μM. The concentrations of this peptide required to inhibit 50% (MIC_50_) and 90% (MIC_90_) of the isolates were both 16 μM, while for polymyxin they were 2 μM (MIC_50_) and 4 μM (MIC_90_). Another relevant point was the bactericidal effect of jelleine-I observed for all isolates. Interestingly, some resistant strains (AC33, AC37) were more sensitive to the bacteriostatic (MIC 8 μM) and bactericidal (CBM 8 μM) effects of jelleine-I than CSAB isolate (ATCC 19606: MIC 16 μM; CBM 16 μM). Therefore, the results indicate that *A. baumannii* isolates with the MDR phenotype may exhibit increased sensitivity to the antibacterial effects of jelleine-I, a finding that is consistent with previous study^24^ highlighting the potential of AMPs to overcome resistance mechanisms that render traditional antibiotics ineffective.

**Table 1:** Minimum Inhibitory Concentration (MIC) and Minimum Bactericidal Concentration (MBC) of Jelleine-I and Polymyxin B against Clinical and Drug-Resistant *A. baumannii* Strains^a^

Strain ID/resistance phenotype	Antimicrobial resistance	Jelleine-I (μM)		Polymyxin B (μM)	
		MIC	MBC	MIC	MBC
AC 03/MDR	β-lactams (including carbapenens), aminoglicosides, quinolones.	16	16	1	1
AC 08/XDR	β-lactams (including carbapenens), aminoglicosides, quinolones.	16	16	2	2
AC 10/PDR	β-lactams (including carbapenens), aminoglicosides, quinolones and polymyxins (B Polymyxin)	32	32	4	4
AC 30/XDR	β-lactams (including carbapenens), aminoglicosides, quinolones, glicilciclines (Tigeciclin) and polymyxins.	16	16	4	4
AC 33/MDR	β-lactams (including carbapenens), aminoglicosides, quinolones.	8	8	1	1
AC 37/PDR	β-lactams (including carbapenens), aminoglicosides, quinolones, glicilciclines (Tigeciclin) and polymyxins.	8	8	8	8
AC 39/PDR	β-lactams (including carbapenens), aminoglicosides, quinolones, glicilciclines (Tigeciclin)	16	16	2	2
AC 40/XDR	β-lactams (including carbapenens), aminoglicosides, quinolones, glicilciclines (Tigeciclin)	16	16	2	2
AC 49/XDR	β-lactams (including carbapenens), aminoglicosides, quinolones.	16	16	0.5	0.5
AC 53/MDR	β-lactams (including carbapenens), aminoglicosides, quinolones.	16	16	1	1
ATCC 19606 (CSAB)	-	16	16	2	2
MIC50		**16**		**2**	
MIC90		**16**		**4**	

a MDR: resistance to at least three classes of antimicrobials; XDR: MDR plus resistance to carbapenems; PDR: XDR plus resistance to polymyxins; CSAB: Carbapenem-susceptible *Acinetobacter baumannii*; MIC50: Concentration required to inhibit 50% of isolates; MIC90: Concentration required to inhibit 90% of isolates.

### Antibacterial Activity of Jelleine-I in Logarithmic Cells

**Figure 4:**
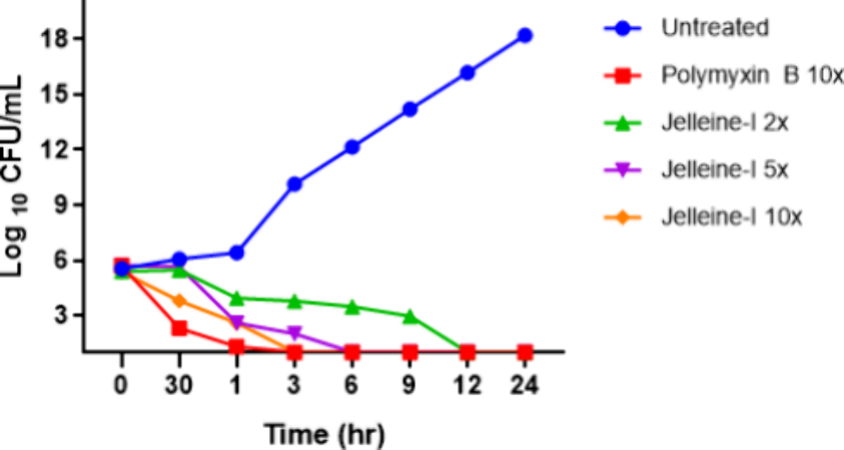
Killing kinetics of jelleine-I and polymyxin against logarithmic cells of CRAB/PDR-*A. baumannii* isolate. The plot displays the number of logarithmic colony-forming units per milliliter (Log10 CFU/mL). Untreated bacterial cells were used as a negative control (blue circles). Polymyxin B was applied as the positive control at 10x MIC (80 μM) (red square). Jelleine-I was tested at concentrations of 2x (16 μM) (green triangle), 5x (40 μM) (purple inverted triangle), and 10x MIC (80 μM) (orange diamond).

The application of *in vitro* models utilizing time-kill curves is an alternative that allows a more detailed evaluation of the pharmacokinetic-pharmacodynamic relationship of an antimicrobial candidate.^25^ For a substance to be considered bactericidal, the number of CFU in the death curve test must be reduced by 3 log10.^26^ To evaluate this effect, the AC37 strain was chosen to represent a more adverse scenario in relation to the antimicrobial resistance profile (resistant to different classes of antimicrobials, including polymyxin) as shown in Table 1. Jelleine-I showed a rapid bactericidal effect against cells in logarithmic growth and was able to completely eliminate the high bacterial load of the *A. baumannii* isolate (CRAB/PDR) within six hours at a concentration five times higher (5x MIC) than the MIC (Figure 4). At twice the MIC, the microbial load was reduced to zero after 12 h. The *A. baumannii* kill kinetics (CRAB/PDR) value of jelleine- I was comparable to that of polymyxin B at the same concentration (10-fold MIC), at which both eliminated the bacterial load from the culture medium after 3 h of incubation. The quick and effective removal of *A. baumannii* demonstrated in this study can help reduce the risk of infectious complications, lower the antimicrobial concentration needed for the desired outcome, decrease the chances of resistance development during clinical use, and shorten the treatment duration.^26^

### Jelleine-I Can Disrupt the Membrane of CRAB Cells Resulting in Cell Lysis

Antimicrobial peptides (AMPs) are known for their ability to selectively interact with the bacterial cell membrane or wall. In general, AMPs have a positive charge and a large amount of hydrophobic residues, which enables their interaction with the negatively charged bacterial membrane.^27^ This interaction facilitates the formation of pores in the membrane, leading to cell lysis and the release of cytoplasmic contents, resulting in a bactericidal effect.^27,28^

**Figure 5:**
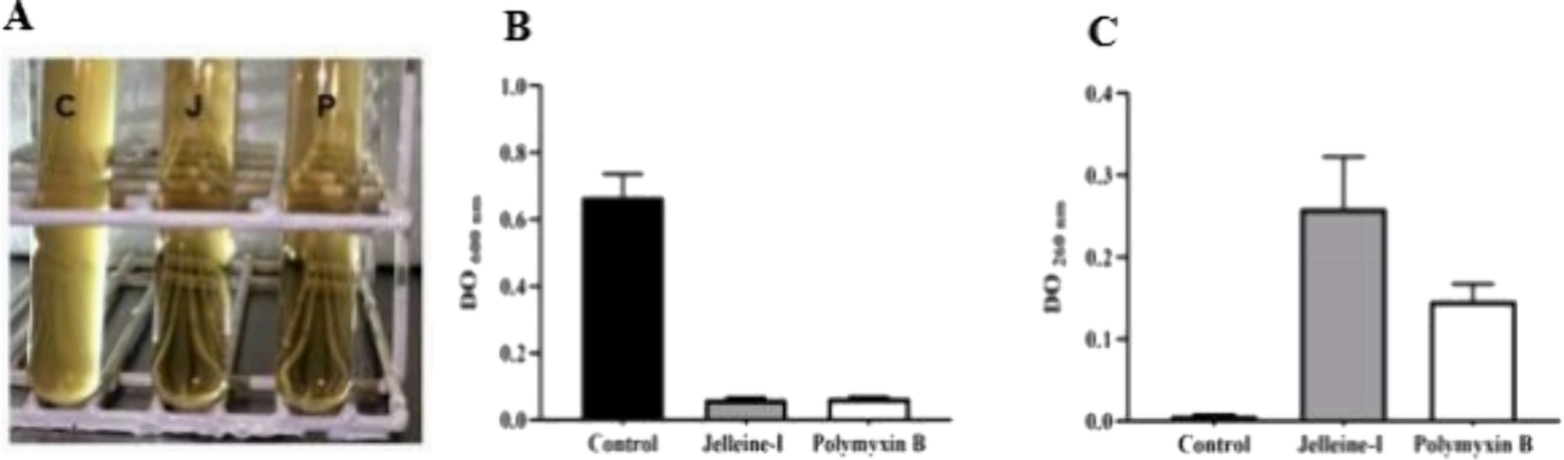
Bacteriolysis. (A and B): Analysis of lysis by visible spectrum (OD 600 nm); C: control; J: jelleine-I; P: polymyxin. Assay of intracellular material with absorption of 260 nm (C). The figures are representative of 3 independent experiments.

The potential of jelleine-I and polymyxin-B to lyse *A.baumannii* cells was assessed by visible and ultraviolet spectrophotometric methods. After incubation of jelleine-I at 10 x MIC (80 μg/mL- AC37 isolate), a significant reduction in the optical density of the treated bacterial suspension was observed relative to the untreated control (Figures 5 A and B). The lytic effect of jelleine-I was comparable to that of polymyxin B. The lysis of PDR-CRAB cells following exposure to jelleine-I was further confirmed by the release of intracellular material that absorbs at 260 nm. As depicted in Figure 5C, treating bacterial suspensions with jelleine-I at 10x MIC for 24 h resulted in an increase in the release of cellular contents, indicating that the peptide may compromise the integrity of the bacterial membrane. These results, added to the studies of ITC, SPR and Hydrodynamic diameter (Dh) and zeta potential (ζ) measurements, reinforce the peptide-membrane interaction and its potential disturbance to the organization of the lipid bilayer, suggesting that this is the main antimicrobial action caused by jelleine-I. Jia et al.^29^ showed that jelleine-I and its derivatives can cause the loss of cell integrity in *E. coli* cells. Jelleine-I initially binds to the microorganism’s cell surface and then disrupts the integrity of the cytoplasmic membrane, resulting in cell death. In the membrane’s amphiphilic environment, the pressure exerted by jelleine-I to accommodate its polar and nonpolar residues can disturb the bilayer’s continuity, causing the vesicle’s contents to leak, which corroborates our results of interaction with the membrane and cell lysis (Figure 5). In addition, peptide may have other action mechanisms such as interaction with genomic DNA, which may justify higher efficacy in resistant phenotypes when compared to strains that are more sensitive.^29^ Polymyxins, on the other hand, under certain conditions, can kill bacteria through other bacterial cell death mechanisms besides cell lysis, such as the production of hydroxyl radicals.^30,31^

**Figure 6:**
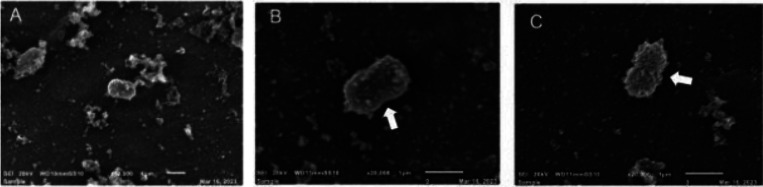
Scanning electron microscopy (SEM) images of cells from *A. baumannii* PDR untreated (A), treated with jelleine-I (B) or polymyxin B (C).

This finding was validated by observing the effect of the peptides on bacterial morphology, as monitored through SEM. In microorganisms treated with jelleine-I, asymmetric divisions and inclusions were observed on the cell surface (Figure 6B – white arrow). The same changes were seen in cells treated with the positive control (Polymyxin B) (Figure 6C – white arrow) and no changes were found in untreated bacteria (Figure 6A).

### Jelleine-I Does Not Present Significant Hemolytic Activity

Although many peptides are active in the membrane of prokaryotes, they seem to be less able to disrupt eukaryotic membranes because there are no negatively charged lipids on the eukaryotic cell surface. However, AMPs can interact with the surface of erythrocytes by binding to sialic acid residues in glycoproteins or glycosphingolipids.^32^ As a result, an increase in hemolysis is observed in most patients. This may be related to various structural factors of the peptides, which also influence antimicrobial efficacy.^33^ The hemolysis percentage of 50% (HD50) was observed at a concentration of 141.9 μM jelleine-I after 1h of incubation. However, this value (141.9 μM) is higher than the MIC value of 8 μM used in the present work. Thus, at the concentrations that showed antibacterial activity of jelleine-I, the results indicate the absence of hemolytic activity. This result is confirmed by Zhou et al.,^34^ who also found high HD50 values for jelleine-I, and Jia et al.^35^ demonstrated that jelleine-I did not induce any significant hemolytic effect, even at high concentrations (256 μg/mL).

### *In Vitro* Cytotoxicity Evaluation

**Figure 7:**
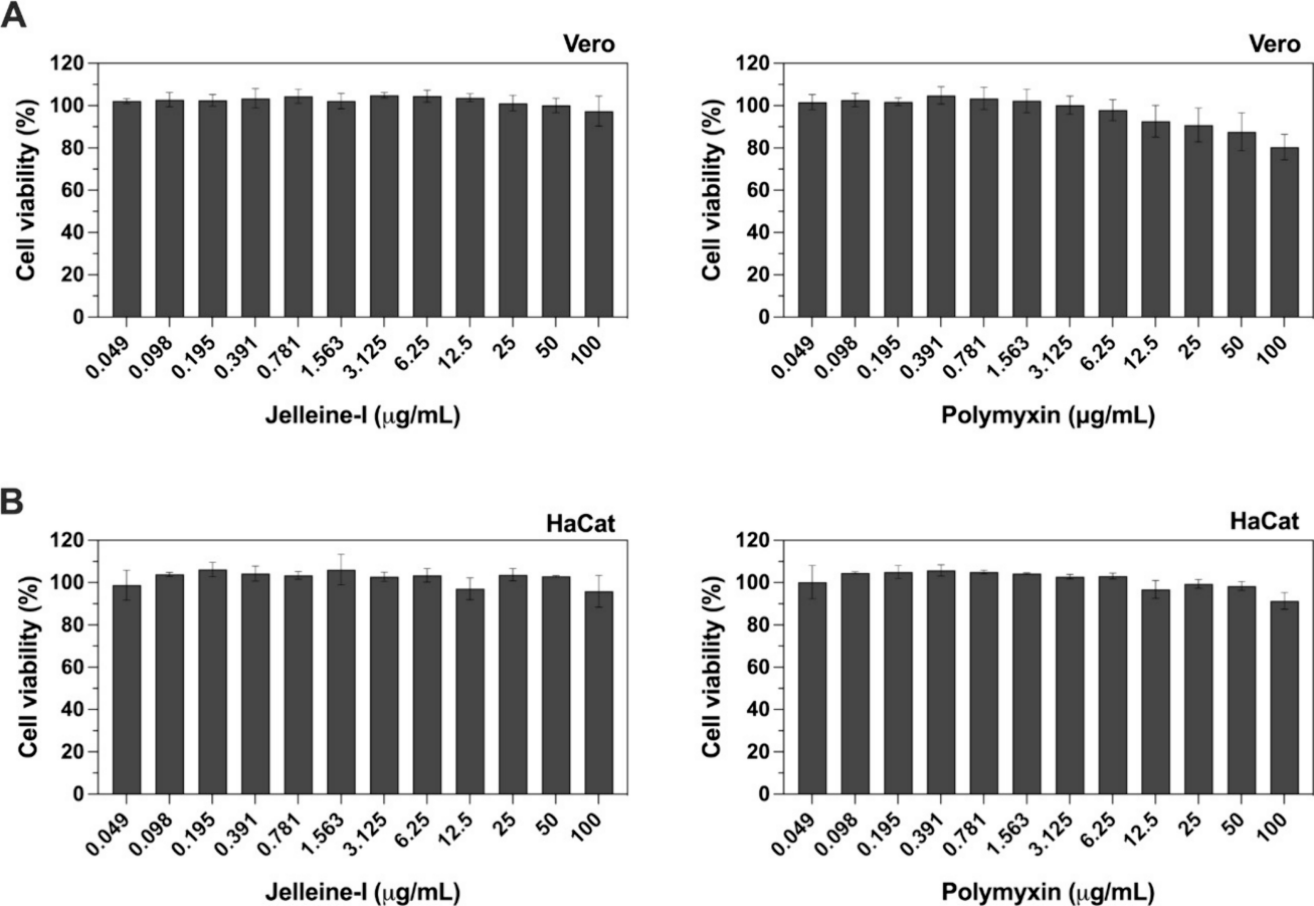
Viability of cells treated with jelleine-I. Concentration–response of VERO (A) and HaCat (B) cells treated with jelleine-I or polymyxin B. Cells were incubated with different concentrations of peptide (0.049 to 100 μg/mL). Cell viability was evaluated after 24 h of incubation using the Alamar Blue reagent and fluorescence values were detected in fluorimeter at 540 nm excitation and 590 nm emission. The values are expressed as the mean ± SD of three independent replicates.

Many antimicrobial candidates fail to be approved for clinical use due to their toxicity. Some of them can bind to host cells, limiting their therapeutic application. Ideal for a AMP is a promising substance that presents high antimicrobial activity and low affinity for human cells, preventing damage to red blood cells and avoiding the rupture of eukaryotic cells. In this study, we explored the potential toxicity of jelleine-I in mammalian cells (VERO and HaCaT) and hemolytic activity in human blood cells. As shown in the Figure 7, the peptide does not significantly reduce cell viability, demonstrating low cytotoxic potential in the cells tested. The results found corroborate Jia et al.^35^ that also found negligible cytotoxicity against mammalian cells types, even at higher tested concentrations of the peptide.

In summary, the AMP jelleine-I, with its short amino acid sequence and minimal cytotoxicity, presents an ideal chemical lead for the development of a novel antimicrobial agent, especially against microorganisms that exhibit high and diverse antimicrobial resistance, such as *A. baumannii*. Our results showed that this peptide has potential antibacterial activity against various clinical phenotypes of this species, which is currently a major health problem, especially in hospitals, with high levels of antimicrobial resistance and mortality.

To our knowledge, this is the first *in vitro* study highlighting the antibacterial activity of jelleine-I against clinical isolates of *A. baumannii*. This could be the first step toward the development of new therapeutic options against this microorganism based on jelleine- I, which has the advantages of low toxicity and potential bactericidal activity.

## Conclusion

Jelleine-I showed promising *in vitro* antibacterial activity against clinical isolates of *A. baumannii* with different levels of resistance (MDR, XDR and PDR) and different clinical origins, with a rapid bactericidal activity (within 3 h after incubation). Regarding the mechanistic effects, the results suggest that this antimicrobial effect could be related to the peptide’s ability to selectively interact with the bacterial cell membrane, leading to bacteriolysis. The results also show that both hemolytic and cytotoxic activities are negligible. However, *in vivo* further studies are needed to fully elucidate the potential of this peptide as a novel antibacterial agent for the treatment of *A. baumannii* infections.

## Materials and Methods

### Materials and Microorganisms

Fmoc-protected L-amino acid derivatives and Fmoc resin (Rink amide, 0.63 mmol-g-^1^) were purchased from Iris Biotech GmbH (Marktredwitz, Germany). Low density polyethylene (LDPE) containers with a capacity of 10 mL were used. Sodium chloride (NaCl), dichloromethane (DCM), dimethylformamide (DMF), diisopropyl ether, ethanol, monobasic sodium phosphate and isopropanol (IPA) were purchased from Química Moderna (São Paulo, Brazil). Acetic anhydride, potassium cyanide (KCN), diisopropylcarbodiimide (DIC), sodium dodecyl sulfate (SDS), Dulbecco’s Modified Eagle Medium (DMEM), phenol, glycerol, 1-hydroxybenzotriazole (HOBt), α-cyano-4-hydroxycinnamic acid (α-cyano), ninhydrin, 4-methylpiperidine (PIPE), pyridine, triisopropylsilane (TIS), tris(2-carboxyethyl)phosphine (TCEP) and Triton X-100 were purchased from Sigma (Saint Louis, MO, USA). AlamarBlue was acquired from Invitrogen, Thermo. Methyl-PEG-Maleimide (mPEG-MAL) was purchased from Polysciences Inc. (Warrington, USA). Acetonitrile and trifluoroacetic acid (TFA) were supplied by J.T. Baker (Center Valley, PA, USA). Peptide calibration standard II was purchased from Bruker Daltonics (Hamburg, Germany). Difco Tryptic Soy Agar (TSA) and Difco Mueller Hinton Broth (MH) were purchased from BD (Sparks, MD, USA). In addition, 1-palmitoyl-2-oleoyl-sn-glycero-3-phosphorylcholine (POPC) and 1-palmitoyl-2-oleoyl-sn-glycero-3-(phospho-rac-(1-glycerol)) (POPG) from Avanti Polar Lipids, Inc. (Alabaster, AL, USA).

*Acinetobacter baumannii* (ATCC 19606) was obtained from the American Type Culture Collection , and 10 CRAB, multidrug-resistant, extensively drug-resistant or pan-drug-resistant *A. baumannii* clinical isolates were obtained from clinical samples of patients treated in 2019 at Hospital João XXIII, Belo Horizonte, Minas Gerais, Brazil. They were previously characterized in terms of their resistance profile to various antibiotics. All isolates were identified by the automated system Vitek 2 (bioMerieux, Hazelwood, MO) and their identity was confirmed with matrix-assisted laser desorption ionization-time-of-flight mass spectrometry (MALDI-TOF MS), with a Microflex LT spectrometer (BrukerDaltonics, MA, USA).

### Synthesis and Purification of Jelleine-I

Peptide synthesis was performed according to the adapted Fmoc protocol,^36^ as described below: 199.7 mg of polymeric resin were weighed, and subsequently was drawn into a syringe of 10.0 mL polypropylene, containing a porous filter. Then, 5.0 mL of DCM were applied into a syringe (resin dilation process) and stirred (450 rpm) for 30 min at room temperature. Next, the DCM was filtered, and the resin was washed. The amino acid couplings were carried out using 4 equiv of Fmoc amino acid derivatives activated by treatment with 1,3-diisopropylcarbodiimide (4 equiv) and 1-hydroxybenzotriazole (4 equiv) in a mixture of N,N-dimethylformamide (DMF):DCM 1:1 (v/v) during 2 h with vortex stirring (450 rpm). Fmoc deprotection was achieved by treatments with a mixture 4-methylpiperidine: DMF 1:4 (v/v) (2 × 15 min, 240 rpm). Coupling and deprotection steps were monitored using the Kaiser test.^36^ For the cleavage of the peptide from the resin, a solution of TFA:H2O:TIS:EDT 95.0:2.5:1.0 (v:v) was drawn into the syringe, which was left under vortex stirring (450 rpm) for 2.5 h. The synthesized product was purified through RP-HPLC using a Shimadzu LC20AD/SPDM20A/RID20 system (Kyoto, Japan) equipped with a Phenomenex C18 semipreparative column (250 mm, 10 mm). The column was equilibrated with a mobile phase composed of 90% phase A (0.1% TFA in water, v/v) and 10% phase B (0.08% TFA in acetonitrile, v/v). Detection was performed at a wavelength of 220 nm. The phase B gradient followed a linear progression: starting at 10% for the first 5 min, increasing to 60% between 5 and 30 min, then rising to 100% from 30 to 45 min, maintaining this level until 50 min, and then decreasing back to 10% from 50 to 60 min.

### MALDI-TOF Characterization

Chromatographic fractions exhibiting the highest absorbance were collected and analyzed using Matrix-Assisted Laser Desorption Ionization – Time of Flight – Mass Spectrometry (MALDI-TOF-MS) with an Auto Flex III mass spectrometer (Bruker Daltonics, Hamburg, Germany). Briefly, the samples were deposited onto a Bruker Daltonics MTP Anchorchip 384 BC (Hamburg, Germany), mixed with a saturated α-cyan solution, and allowed to dry at room temperature. The analysis was performed using the Pepmix calibration standard (up to 4 kDa),^37^ and mass spectra (MS) were recorded in positive mode after calibration with the peptide calibration standard.^37^ Spectra were generated and analyzed using Mass Data Miner software.^37^ MALDI-TOF analyzes were performed at the Laboratory of the Proteomics and Arachnids Service, Research Directorate, Fundação Ezequiel Dias (FUNED).

MALDI-TOF-MS and MALDI-TOF-MS-MS spectra of purified jelleine-I were also obtained in an Autofle III SmartBeam spectrometer (Bruker Daltonics, Germany). These experiments were performed at the LMProt facilities available at CELAM (UFMG).

### Characterization by NMR Spectroscopy

NMR experiments were performed for jelleine-I at 1.3 mM in DMSO-*d*6. Tethamethylsilane was used as internal reference. The experiments were performed at 25 °C on a Bruker Avance Neo 600 spectrometer equipped with a 5 mm multinuclear TXI probe. Total Correlation Spectroscopy (TOCSY) spectra were acquired using a MLEV- 17^37^ pulse sequence with a spin-lock time of 90 ms. Nuclear Overhauser Spectroscopy (NOESY) spectra^38^ were acquired using different mixing times to check for spin diffusion (150, 200, and 250 ms). The following parameters were used for the acquisition of TOCSY and NOESY spectra: spectral width of 10870 Hz, 512 t1 increments were collected with 16 transients of 2048 points. ^1^H–^13^C Heteronuclear Single Quantum Coherence (HSQC) spectra were acquired with F1 and F2 spectral widths of 31695 and 10870 Hz, using 256 t1 increments with 32 transients of 2048 points for each free induction decay. ^1^H–^13^C HSQC was acquired in an edited mode so the CH and CH3 correlations show positive phase whereas CH2 correlations show negative phase.^39^^1^H- ^15^N HSQC spectra were acquired using F1 and F2 spectral widths of 3041 and 10870 Hz, 80 t1 increments were collected with 400 transients of 2048 points.^39^

### Preparation of Large Unilamellar Vesicles (LUVs)

LUVs were prepared using the phospholipids 1-palmitoyl-2-oleoyl-sn-glycero-3-phosphocholine (POPC) and 1-palmitoyl-2-oleoyl-sn-glycero-3-phospho-(1′-rac-glycerol) (POPG) for Surface Plasmon Resonance (SPR), Dynamic Light Scattering (DLS), and Zeta Potential Measurements. The phospholipids were transferred to a glass tube while maintaining a molar ratio of POPC:POPG of 3:1 and dissolved in 2 mL of chloroform at room temperature. The organic solvent was removed with a rotary evaporator, forming a lipid film. The dried lipid film was then hydrated with 2 mL of 100 mM Tris-HCl buffer (pH 7.5) containing 100 mM NaCl at 35 °C to form a 20 mM stock solution. The resulting multilamellar vesicles (MLVs) were subjected to five freeze–thaw cycles, alternating between liquid nitrogen and a water bath at 35 °C. Subsequently, the MLVs were extruded using a 10 mL stainless steel extruder (Lipex Biomembranes Inc., Vancouver, Canada) at approximately 35 °C to obtain LUVs with an average diameter of 100 nm. The total lipid concentration of the extruded LUVs was determined using a colorimetric method as described by Stewart.^40,41^

### Isothermal Titration Calorimetry (ITC)

ITC analyzes were performed in a Malvern VP-ITC microcalorimeter (Worcestershire, United Kingdom) at 25 °C in the Laboratory for Synthesis and Structure of Biomolecules (LASEB - UFVJM), as previously described by Brito et al.^17^

### Dynamic Light Scattering (DLS) and Zeta Potential (ζ-potential)

A Z98 Zetasizer Nano ZS Malvern BI-900 particle analyzer (Worcestershire, UK) was used to measure the changes in hydrodynamic diameter (Dh) and zeta potential (ζ-potential) of 500 μM POPC:POPG LUVs following the addition of peptides at 35 °C. A monochromatic 4 mW Ne laser (λ = 633 nm) was used to detect light scattering at a 90° angle. Ten different LUV samples with different peptide concentrations were used in the triplicate experiments, which were prepared with a final volume of 800 μL. Each sample consisted of a 1 mM POPC:POPG LUVs stock solution and a 2 mM peptide solution suspended in a 10 mM acetate buffer (pH 5.6) containing 50 mM NaCl. Table 2 shows the composition of the individual samples. After each injection, a 25 min stabilization interval was inserted before measuring the hydrodynamic diameter and zeta potential.

**Table 2:** Sample Composition Used for Hydrodynamic Diameter (*D*h) and Zeta Potential (ζP) Measurements^a^

**Sample**	*Vpep***(**μ**L)**	*VLUVs***(**μ**L)**	*Vbuffer***(**μ**L)**
1	0	400	400
2	2	400	398
3	4	400	396
4	6	400	394
5	8	400	392
6	12	400	388
7	16	400	384
8	20	400	380
9	25	400	375
10	30	400	370

a *V*pep = volume of 2 mM peptide stock solution; *V*LUVs = volume of 1 mM POPC:POPG LUVs stock solution and *V*buffer = volume of 10 mM acetate buffer solution (pH 5.6).

#### Surface Plasmon Resonance (SPR)

SPR measurements were performed as previously described by Brito et al.^17^ with minor changes. At 35 °C at a flux of 10 μL·min^–1^ for LUV immobilization and recorded at 850 nm using an SPR Navi 200 instrument (BioNavis Ltd., Ylöjärvi, Finland). Measurements were performed in triplicate using angular scan mode (40–78 degrees), with SPR curves recorded every 3.5 s. SPR gold sensor chips were previously functionalized with DL-dithiothreitol (DTT) as described elsewhere.^14^ DTT-gold chips were used for phospholipid immobilization, washed with successive 5 min injections (50 μM.min^–1^) of 5% Hellmanex III (Sigma, St Louis, MO), 2-propanol, and Milli-Q water, *in situ* in the flow channel, immediately before each experiment. For each measurement, the sensor chip was first exposed to the running buffer (10 mM acetate buffer, pH 5.6, 50 μM.min^–1^) and then 50–250 μM POPC:POPG LUVs for approximately 13 min (10 μM.min^–1^) until the baseline stabilized. Experiments consisted of 50 μL injections of 10 μM peptide solution in running buffer (10 mM acetate pH 5.6). The surface partition coefficient (*K*) of peptide-membrane interactions was determined from SPR experiments by fitting the data to the eq 1, considering the electrostatic interaction:^42,43^
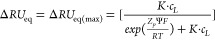
1Where Δ*RU*eq(max) is the observable *RU*eq intensity 12 min after peptide injection (∼32 min experimental time) normalized to the stabilized *RU*eq intensity of the LUV suspension in the absence of the peptide (∼15 min experimental time), *Zp* is the charge on the peptide, Ψ is the membrane surface potential, *F* is Faraday’s constant, *R* is the universal gas constant, and *T* is temperature. The concentration of accessible lipid (considering the outer leaflet of the membrane bilayer, which accounts for 60% of the total lipid concentration) is represented by *cL*. Quantification of the total phospholipid content immobilized in the sensor chip was performed as described by Junior et al.^16^ To determine the phospholipid content in LUVs solutions, the phospholipid stock suspension and the collected volume (within 5 min following each injection) were quantified. These samples were mixed with 1.2 mL of ammonium ferrothiocyanate aqueous solution and 1.5 mL of chloroform. The resulting biphasic system was vigorously shaken for 1 min. The lower chloroform phase was carefully extracted using a glass pipet and transferred to a separate tube. Anhydrous sodium sulfate was then added to remove residual water before analysis. Phospholipid quantification was performed using a Varian Cary 50 UV–vis spectrophotometer (Walnut Creek, CA, USA).

The membrane surface potential, Ψ, was calculated from the ζ-potential and considering the exponential decay of the electrostatic potential,^44,45^ according to the following formula:
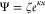
2

Here, ζ stands for the ζ -potential, κ for the inverse of the Debye length, and *x* for the hydrodynamic layer thickness, which in this case is assumed to be *x* = 0.25 nm from the POPC:POPG LUV surface.^46,47,48^ The ζ -potentials of 0.5 mM POPC:POPG LUVs were measured at 25 °C in a Malvern Zetasizer Nano ZS particle analyzer (Malvern Instrument Ltd., Worcestershire, UK) as described by Junior et al.^16^ The ζ -potential value used in Equation (−34.7 ± 0.1 mV) corresponds to the average of the values of three LUV solutions.

### Antimicrobial Activity

#### Minimal Inhibitory Concentration (MIC) and Minimal Bactericidal Concentration (MBC) Evaluation

To determine the MIC values for peptides, Mueller-Hinton (MH) broth supplemented with 0.002% Tween-80 was used, following the broth microdilution method as per the Clinical and Laboratory Standards Institute guidelines. After 24 h of incubation at 37 °C under aerobic conditions, colonies from different *A. baumannii* isolates were collected with an inoculation loop and transferred to a 0.9% (m/v) NaCl saline solution. The inoculums were checked in a spectrophotometer at 625 nm; colonies were added until an absorbance corresponding to 1.0 × 10^8^ CFU/mL was obtained. Then, 50 μL of resultant suspension was transferred to 10 mL of Mueller-Hinton broth (MHB; Himedia, Mumbai, MH, India) resulting in an inoculum of 1.0x 10^6^ CFU/mL, which was employed in the posterior experiments. MIC assays were performed in microdilution plates using inoculums of 10^5^ bacterial cells per well and concentrations of peptide ranging from 64 to 0.50 μM. All analyses were performed in triplicate. Positive (growing control - MH + bacterial inoculum), negative (sterility control - MH + saline solution) and antimicrobial (MH + antimicrobial) controls were included in each batch of tests. Additionally, the minimal bactericidal concentration (MBC) was determined by plating 10 μL from the optically clear wells of the MIC assay onto Mueller-Hinton agar (MHA). Following incubation for 24 h at 35 ± 2 °C, the MBC was defined as the lowest compound concentration that eliminated at least 99.9% of the initial inoculum compared to the untreated control. The specific values of molar peptide concentration used in this work were estimated according to the method described by Schmid.^49^

#### Time-Kill Curve

A preinoculum of CRAB with 10^8^ CFU/mL was first prepared as described in section 2.8.1. Then 50 μL of the preinoculum was added to test tubes containing 10 mL of Mueller-Hinton broth (MHB) (Himedia, Mumbai, MH, India). The bacteria were then treated with jelleine-I at concentrations of 2x, 5x and 10x MIC. Untreated cells and polymyxin at 10x MIC concentration were used as controls. The tubes were incubated at 37 °C with an aeration rate of 225 rpm. At different time points (0, 0.5, 1, 3, 6, 12, or 24 h), 100 μL of the sample was serially diluted (10-^1^ to 10-^5^) in sterile saline (0.9% NaCl; Synth, São Paulo, SP, Brazil) and plated on Mueller-Hinton Agar (MHA) (Himedia, Mumbai, MH, India). The plates were incubated for 24 h and the CFU/ml was determined.^49^

#### Assessment of Bacterial Lysis

The potential of jelleine-I and polymyxin B to induce lysis of XDR-*A. baumannii* cells was evaluated by both visible and ultraviolet spectrophotometric methods. For lysis analysis via the visible spectrum, 10 mL of bacterial culture with an optical density at 600 nm (OD_600_) of 1.0 was treated with 10 × MIC of either jelleine-I or polymyxin for 24 h. After incubation, each culture was transferred to glass test tubes and photographed.^50^ The OD_600_ of the bacterial suspensions after treatment was also measured.

For ultraviolet lysis analysis, the release of intracellular material was quantified with absorbance at 260 nm using a modified version of the method of Bruin and Birnboim.^51^ Aliquots of 5 mL bacterial suspensions (10^8^ CFU/mL) in sterile saline were treated with jelleine-I or polymyxin B at 10x MIC for 24 h. Following treatment, the cells were centrifuged at 2,500 × g for 5 min, and the absorbance of the supernatant at 260 nm was measured using an ultraviolet spectrophotometer (Hitachi U1100, Lancashire, UK).

#### Scanning Electron Microscopy (SEM)

To evaluate the morphological changes induced by jelleine-I or polymyxin B on XDR-*A. baumannii* cells, scanning electron microscopy (SEM) analysis was performed according to the method of Ravensdale et al.^52^ The samples to be analyzed were mounted on stubs and coated with a 10 nm thick gold layer by sputtering. The samples were then visualized using a scanning electron microscope (Jeol JSM-6010Plus/LA, Germany).

#### Hemolytic Activity Determination

The hemolytic activity of jelleine-I against human erythrocytes was evaluated using the previously reported methodology.^53^ In brief, fresh blood was drawn from a healthy volunteer and placed in heparin-sodium anticoagulation tubes. After the human erythrocytes were separated from the blood by centrifugation at 300 g for 5 min, they were rinsed three times with five milliliters of phosphate-buffered saline (PBS). The concentrations of the peptide solutions that were prepared ranged from 256 μM to 1 μM. Each well of a 96-well microtiter plate received 100 μL of 8% erythrocytes to perform the experiment. The final peptide concentrations were between 256 μM and 1 μM after 100 μL of the peptide solution was added to each well. After 1 h of incubation at 37 °C, the plate was centrifuged at 300 g for 5 min. The supernatants from each well were transferred to a new sterile 96-well microplate and the release of hemoglobin was monitored using a plate reader (Multiskan FC – ThermoScientific, Osterode am Harz, Germany) at 405 nm. Independent experiments were conducted in triplicate. Triton X-100 (1%) and PBS were used as positive and negative controls, respectively.

#### *In Vitro* Cytotoxicity Evaluation

The *in vitro* cytotoxicity of the peptide to kidney epithelial cells of *Cercopithecus aethiops* (VERO ATCC CCL81) and human keratinocytes (HaCaT) was evaluated using AlamarBlue reagent.^54^ In summary, the cells were cultured and seeded in a 96-well tissue culture plate at a density of 1.0 × 10^4^ cells per well in DMEM supplemented with 10% fetal bovine serum, 2 mM l-glutamine, and 1% penicillin/streptomycin (Gibco). The cells were incubated at 37 °C in a 5% CO_2_ atmosphere, and when they reached approximately 80% confluence, they were treated with the peptide or a control (polymyxin B) at serial concentrations. After 24 h of incubation, the medium was replaced with DMEM containing 10% AlamarBlue (Invitrogen), and the plate was incubated for an additional 3 h at 37 °C. Fluorescence measurements were taken using a Cytation 5 cell imaging (Biotek) fluorimeter, with excitation at 540 nm and emission at 590 nm. Three independent replicates were performed, and the average values were used for subsequent analyses.

## Supporting Information Available

Fractions of the chromatographic peaks verified by matrix-assisted laser distribution ionization–time of flight–mass spectrometry (MALDI-ToF-MS); mass spectra of the purified peptide and the ^1^H–^15^N HSQC spectrum of jelleine-I at 2.0 mM in DMSO-*d*_6_; certain regions of the ^1^H–^13^C HSQC spectrum of jelleine-I in DMSO-*d*_6_; region corresponding to the amide-aliphatic side chains in the TOCSY spectrum of jelleine-I in DMSO-*d*_6_ (PDF)

## Abbreviations

WHO: World Health Organization
AMR: Antimicrobial resistance
CRAB: Carbapenem-resistant strains of *Acinetobacter baumanni*
PDR: Pan-resistance
XDR: Plus resistance to polymyxins
AMPs: Antimicrobial peptides
LUVs: Large unilamellar vesicles
POPC: 2-Oleoil-1-pamlitoil- sn -glicerol-3-fosfocolina
POPG: 2-Oleoil-1-pamlitoil- sn - glicerol-3-glicerol
MH: Mueller-Hinton
MHA: Mueller-Hinton agar
MHB: Mueller- Hinton broth
MIC: Minimal inhibitory concentration
MBC: Minimal bactericidal concentration
