# Supplementary material for: Jelleine-I Membrane Interaction-related Biological Properties and Antimicrobial Activity against MDR, XDR, and PDR-Acinetobacter baumannii Clinical Isolates
Source: ACS Omega. 2025 Mar 11;10(11):10938–48. doi: 10.1021/acsomega.4c09073 (PMC11947796; doi:10.1021/acsomega.4c09073)
Supplement: Supplementary file 1 — ao4c09073_si_001.pdf [file ao4c09073_si_001.pdf]

Supporting information

Jelleine-I membrane interaction-related biological properties and antimicrobial activity against MDR, XDR and PDR-*Acinetobacter baumannii* clinical isolates

Adrielle Pieve de Castro[1*], Julio Cesar Moreira Brito[2], Simone Odília Antunes Fernandes[1], Wanderson Aparecido Brandão Candido[3], Amanda Souza Félix[3], Rodrigo Moreira Verly[3], Jarbas Magalhães Resende[4], Letícia Lopes-de-Souza[5], Carlos Chávez- Olórtegui[5], Valbert Nascimento Cardoso[1]

[1] Laboratório de Radioisótopos, Departamento de Análises Clínicas e Toxicológicas, Faculdade de Farmácia, Campus Pampulha, 31270-901, Universidade Federal de Minas Gerais, Belo Horizonte, Minas Gerais, Brazil.

[2] Fundação Ezequiel Dias; Diretoria de Pesquisa e Desenvolvimento, 30510-010, Belo Horizonte, MG, Brazil.

[3] Departamento de Química, Faculdade de Ciências Exatas; Universidade Federal dos Vales do Jequitinhonha e Mucuri, 39100-000, Diamantina, MG, Brazil.

[4] Departamento de Química, Instituto de Ciências Exatas; Universidade Federal de Minas Gerais, 31270-901, Belo Horizonte, MG, Brazil.

[5] Departamento de Bioquímica e Imunologia, Instituto de Ciências Biológicas, Universidade Federal de Minas Gerais, 31270-901, Belo Horizonte, MG, Brazil.

*Corresponding author
Adrielle Pieve de Castro – Faculdade de Farmácia, Universidade Federal de Minas Gerais, Av. Presidente Antônio Carlos 6627 Pampulha, 31270-901 Belo Horizonte, MG, Brazil. Tel.: +55 35 9889-1217. E-mail address: adriellepieve@hotmail.com.

Supporting information

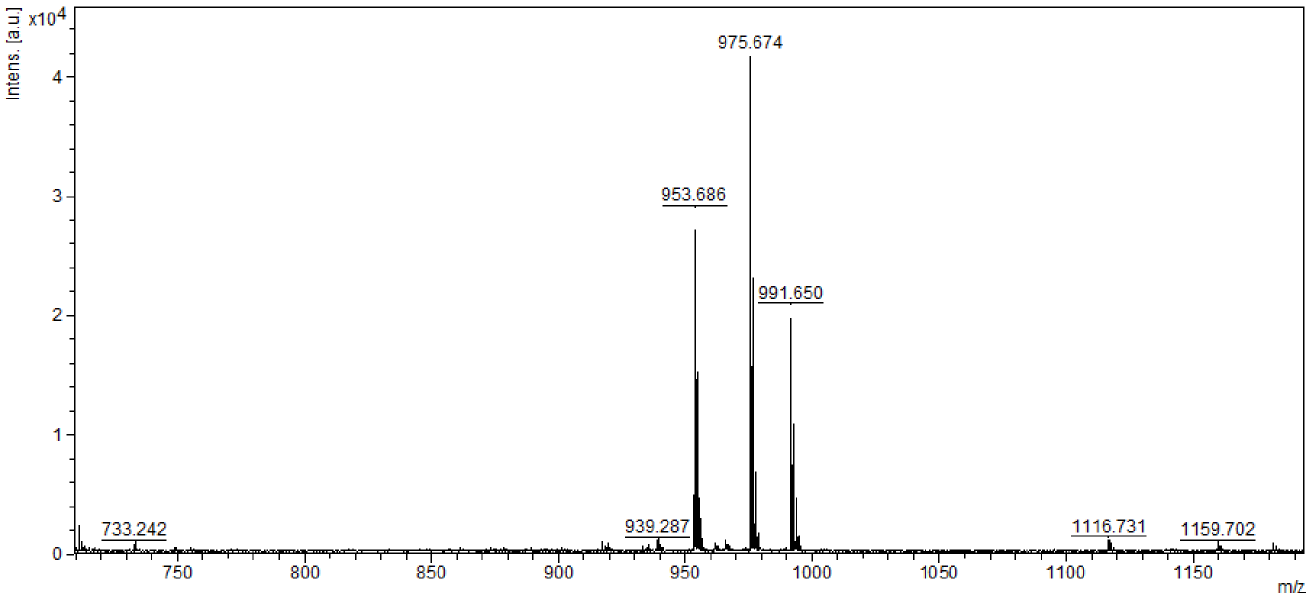

Figure S1. The fractions of chromatographic peaks with the largest absorbance were collected and analyzed by Matrix-Assisted Laser Desorption Ionization – Time of Flight – Mass Spectrometry (MALDI-ToF-MS) using an Auto Flex III (Bruker Daltonics, Hamburg, Germany) mass spectrometer.

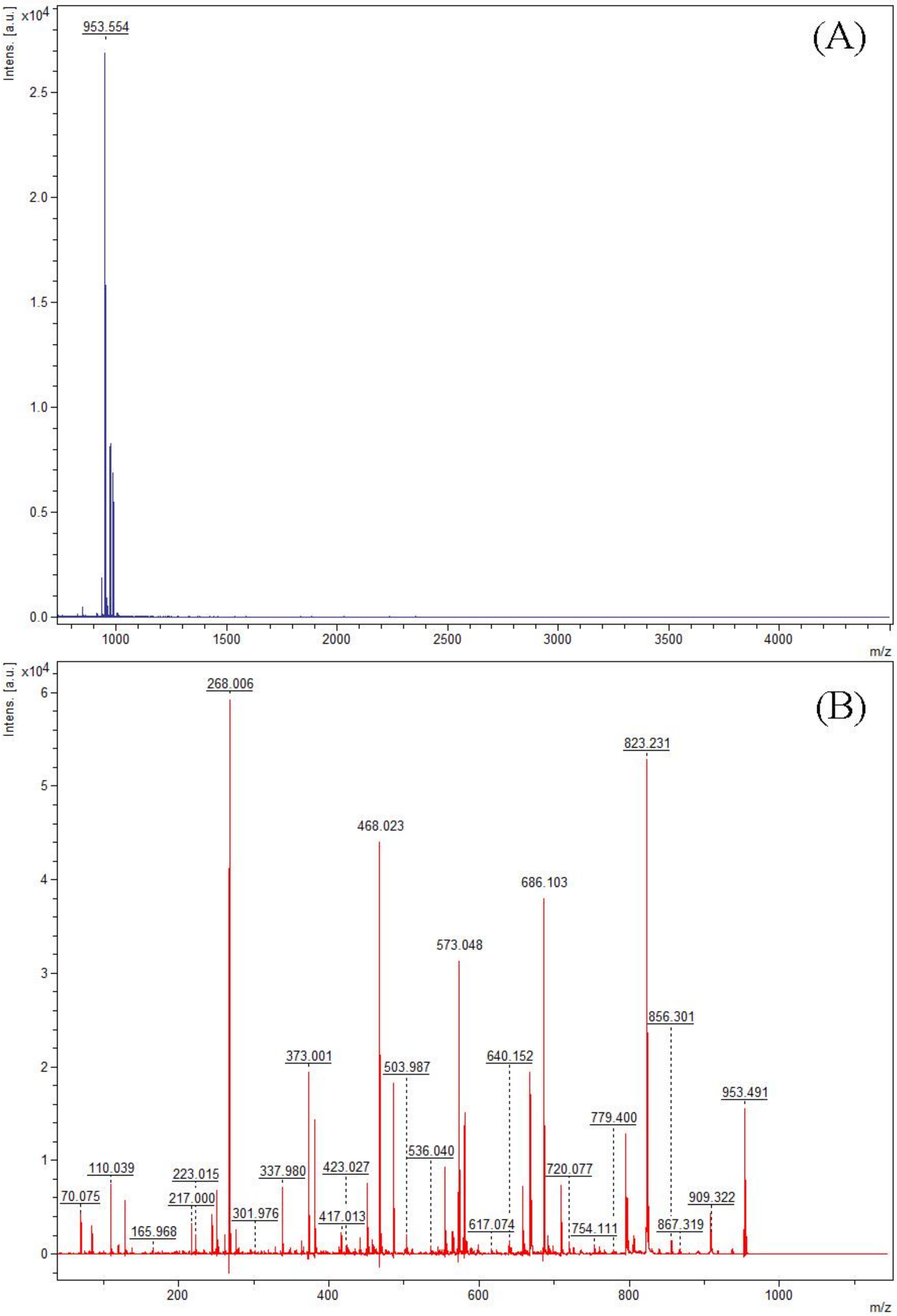

Figure S2. Mass spectra of purified Jelleine-I. (A) MALDI-TOF-MS spectrum with the observed [M+H$^+$] at m/z 953.554 (theoretical value 953.577). (B) MALDI-TOF-MS-MS spectrum with the [M+H$^+$] signals of the positively charged PFKISIHL-NH_2 (953.491), PFKISIH (823.231), PFKISI (686.103), PFKIS (573.048), PFKI (486.023), PFK (373.001) and PF (268.000), corresponding to the y-ions b2, b3, b4, b5, b6, b7 and b8, respectively. The iminium ions of P and H were detected at m/z 70.075 and 110.039, respectively.

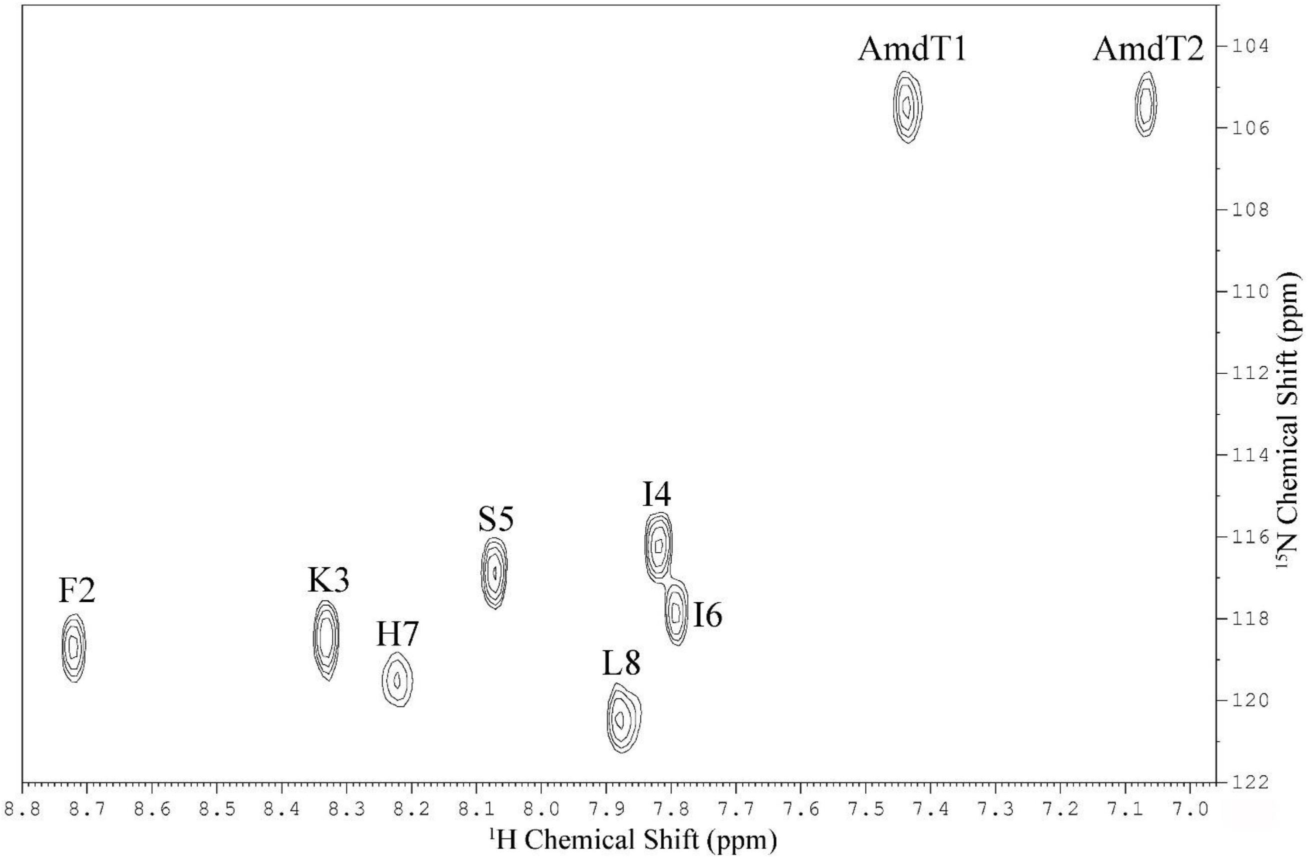

Figure S3. ^{1}H-^{15}N HSQC spectrum of Jelleine-I at 2.0 mM in DMSO-d_6, 25 ^{o}C. All H–N correlations related to the formed peptide bonds are indicated, as well as the H–N correlations of the C-terminal carboxamide.

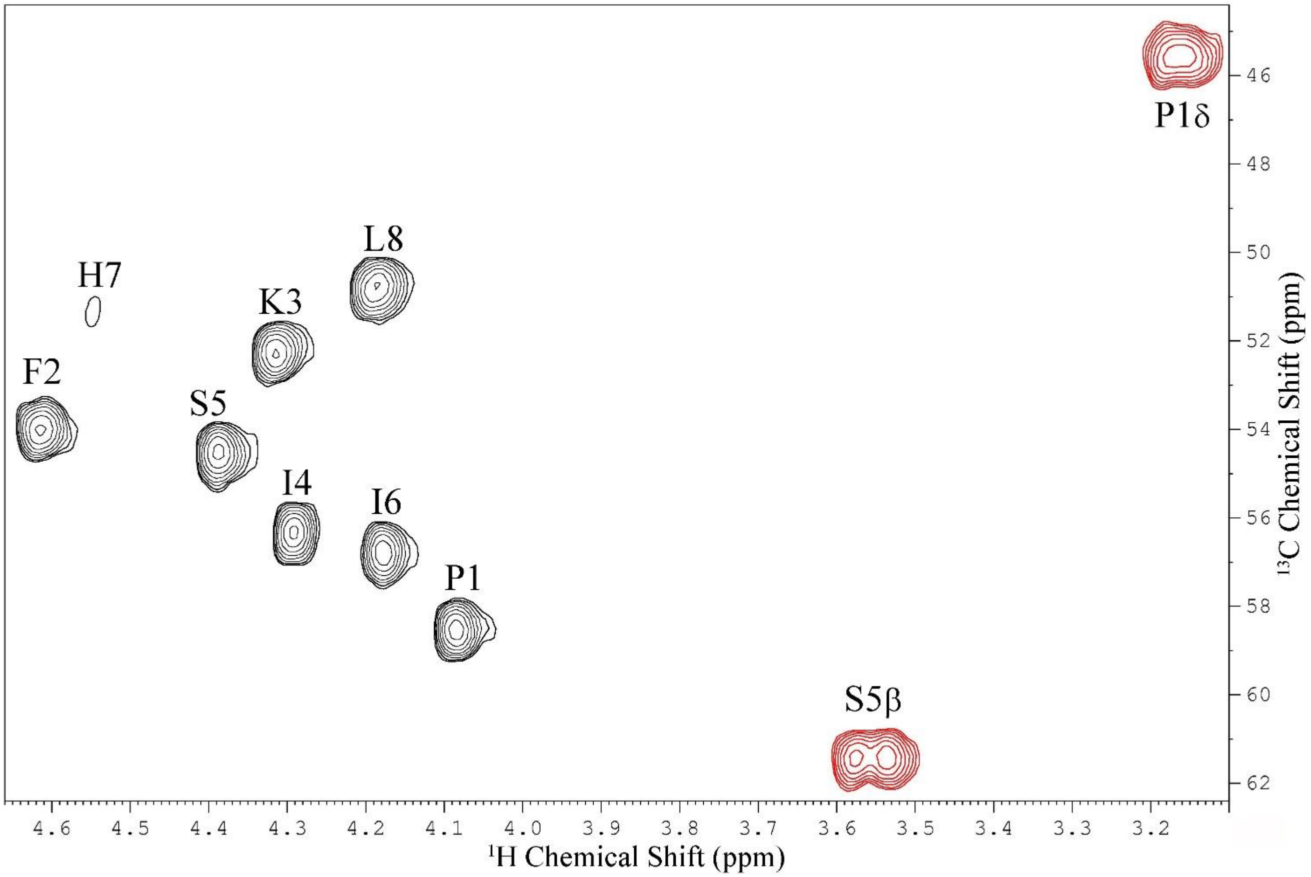

Figure S4. Selected spectral region of the ^{1}H-^{13}C HSQC spectrum of Jelleine-I in DMSO-d_6, 25 °C. The spectrum was acquired in an edited mode in such a way that CH and CH_3 correlations show positive phase (correlations in black), whereas CH_2 correlations show negative phase (correlations in black) [1]. All Hα– Cα correlations are presented, as well the Hβ– Cβ correlation of Ser-5 and Hδ–Cδ correlation of Pro-1, which are characterized by relatively high chemical shifts of aliphatic side chain carbons.

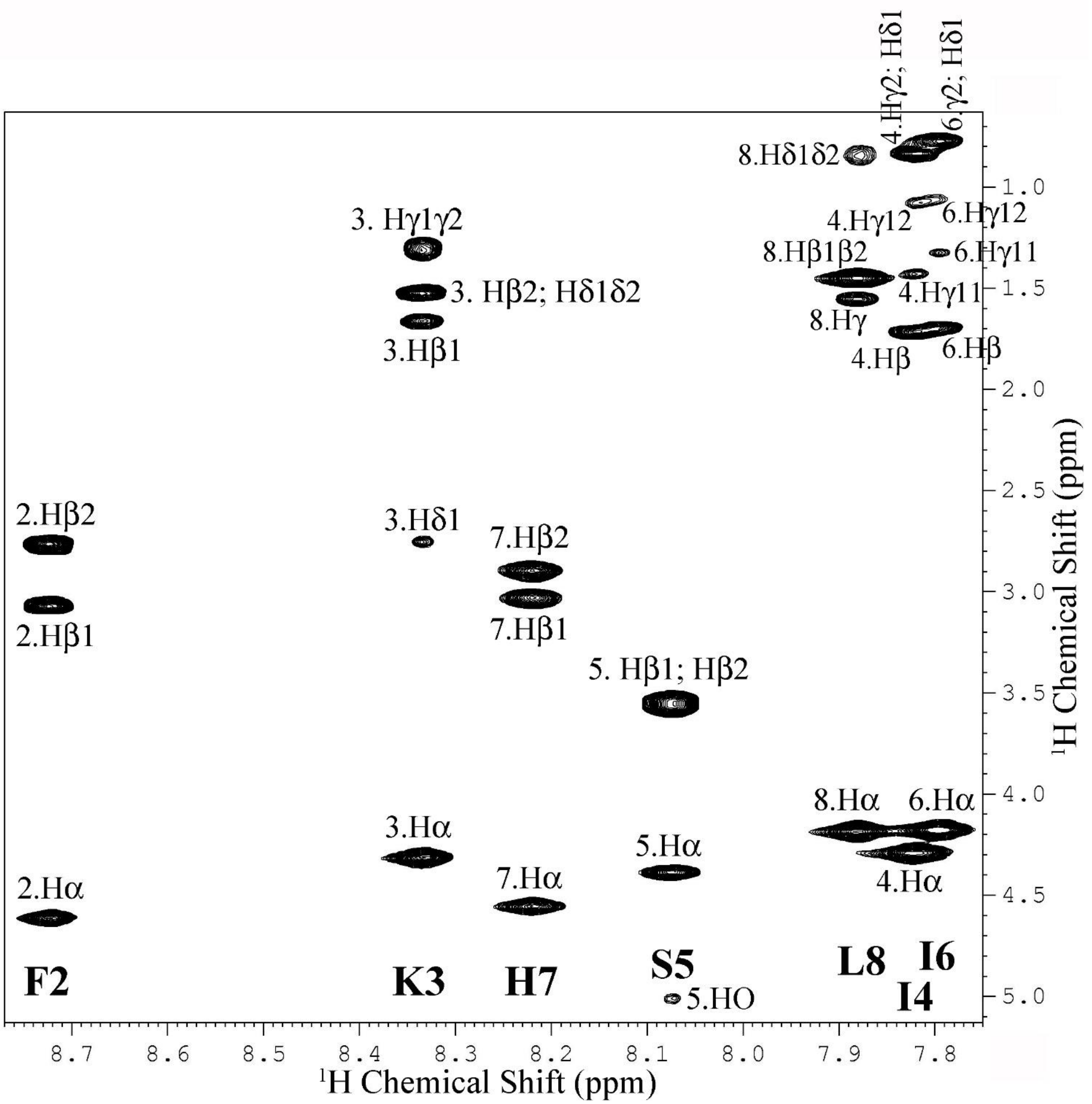

Figure S5. Amide - aliphatic side chain region of the TOCSY spectrum of Jelleine-I in DMSO-d_6, 25 °C. The characteristic spin system detected from the amide ^{1}H chemical shift of each amino acid residue is indicated.

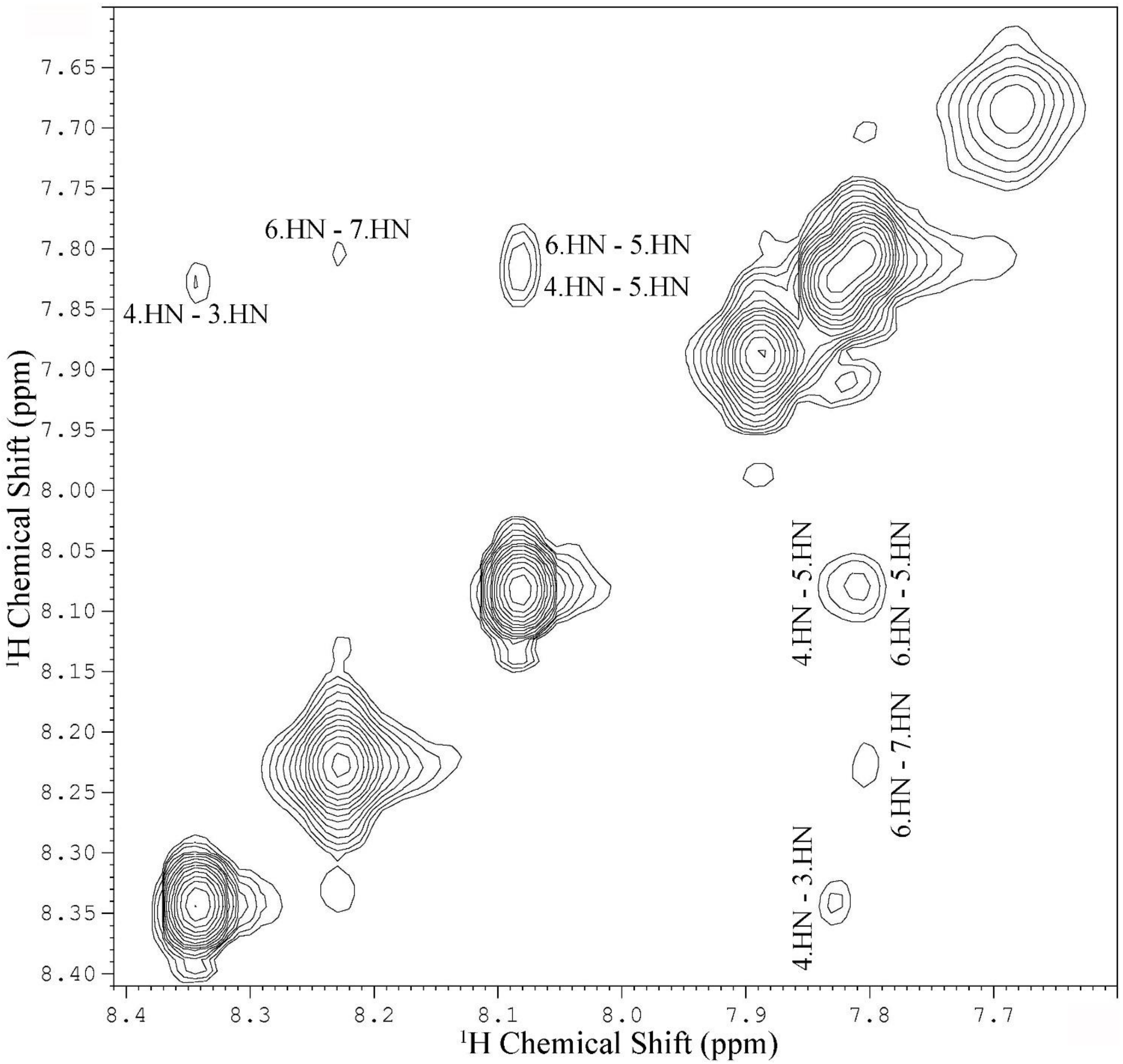

Figure S6. Partial NOESY spectrum of Jelleine-I in DMSO-d_6, 25 °C. The inter-residue correlations between the amide hydrogens of Ile-4 & Lys-3 (4.HN - 3.HN) and of Ile-6 & His-7 (6.HN - 7.HN) were used to unequivocally attribute the spin systems of the two isoleucine residues.

2 Reference

1. Willker, W., Leibfritz, D., Kerssebaum, R., Bermel, W. (1993). Gradient selection in inverse heteronuclear correlation spectroscopy, *Magnetic Resonance in Chemistry*, *31*(3), 287–292. https://doi.org/10.1002/mrc.1260310315
